# Immunogenicity, reactogenicity and safety of an inactivated quadrivalent influenza vaccine candidate versus inactivated trivalent influenza vaccine: a phase III, randomized trial in adults aged ≥18 years

**DOI:** 10.1186/1471-2334-13-343

**Published:** 2013-07-24

**Authors:** Dorothee Kieninger, Eric Sheldon, Wen-Yuan Lin, Chong-Jen Yu, Jose M Bayas, Julian J Gabor, Meral Esen, Jose Luis Fernandez Roure, Silvia Narejos Perez, Carmen Alvarez Sanchez, Yang Feng, Carine Claeys, Mathieu Peeters, Bruce L Innis, Varsha Jain

**Affiliations:** 1 Zentrum für Kinder- und Jugendmedizin, Universitätsmedizin, Mainz, Germany; 2 Miami Research Associates, Miami, USA; 3 Department of Family Medicine, School of Medicine, China Medical University, and China Medical University Hospital, Taichung, Taiwan; 4 Department of Internal Medicine, National Taiwan University, Taipei, Taiwan; 5 Adult Vaccination Center, Preventive Medicine and Epidemiology Unit, Hospital Clínic de Barcelona, Barcelona, Spain; 6 Institute of Tropical Medicine, University of Tübingen, Tübingen, Germany; 7 Àrea Bàsica de Salut La Roca del Vallès, Barcelona, Spain; 8 Cap Centelles, Barcelona, Spain; 9 CAP Balenyà/ABS Centelles, Barcelona, Spain; 10 GlaxoSmithKline Vaccines, Wavre, Belgium; 11 GlaxoSmithKline Vaccines, King of Prussia, USA

**Keywords:** Non-inferiority, Quadrivalent, Seasonal influenza, Superiority, Trivalent

## Abstract

**Background:**

Two antigenically distinct influenza B lineages have co-circulated since the 1980s, yet inactivated trivalent influenza vaccines (TIVs) include strains of influenza A/H1N1, A/H3N2, and only one influenza B from either the Victoria or Yamagata lineage. This means that exposure to B-lineage viruses mismatched to the TIV is frequent, reducing vaccine protection. Formulations including both influenza B lineages could improve protection against circulating influenza B viruses. We assessed a candidate inactivated quadrivalent influenza vaccine (QIV) containing both B lineages versus TIV in adults in stable health.

**Methods:**

A total of 4659 adults were randomized 5:5:5:5:3 to receive one dose of QIV (one of three lots) or a TIV containing either a B/Victoria or B/Yamagata strain. Hemagglutination-inhibition assays were performed pre-vaccination and 21-days after vaccination. Lot-to-lot consistency of QIV was assessed based on geometric mean titers (GMT). For QIV versus TIV, non-inferiority against the three shared strains was demonstrated if the 95% confidence interval (CI) upper limit for the GMT ratio was ≤1.5 and for the seroconversion difference was ≤10.0%; superiority of QIV versus TIV for the alternate B lineage was demonstrated if the 95% CI lower limit for the GMT ratio was > 1.0 and for the seroconversion difference was > 0%. Reactogenicity and safety profile of each vaccine were assessed. Clinicaltrials.gov: NCT01204671.

**Results:**

Consistent immunogenicity was demonstrated for the three QIV lots. QIV was non-inferior to TIV for the shared vaccine strains, and was superior for the added alternate-lineage B strains. QIV elicited robust immune responses against all four vaccine strains; the seroconversion rates were 77.5% (A/H1N1), 71.5% (A/H3N2), 58.1% (B/Victoria), and 61.7% (B/Yamagata). The reactogenicity and safety profile of QIV was consistent with TIV.

**Conclusions:**

QIV provided superior immunogenicity for the additional B strain compared with TIV, without interfering with antibody responses to the three shared antigens. The additional antigen did not appear to alter the safety profile of QIV compared with TIV. This suggests that the candidate QIV is a viable alternative to TIV for use in adults, and could potentially improve protection against influenza B.

**Trial registration:**

Clinical Trials.gov: NCT01204671/114269

## Background

Despite widespread vaccination programs, influenza remains a major cause of hospitalization and death in adults, particularly among older adults and those with chronic illnesses [1]. Among healthy adults, influenza is an important cause of outpatient medical visits and worker absenteeism, burdening health care systems and leading to substantial societal costs [2-4].

Inactivated trivalent influenza vaccines (TIVs), used for vaccination against seasonal influenza, include two influenza A strains (A/H1N1, A/H3N2) and one influenza B strain, selected using surveillance-based forecasts [5]. Two antigenically distinct influenza B lineages (B/Yamagata and B/Victoria) emerged globally in humans in the early 1980s, and have co-circulated in the US since 2000. Furthermore, vaccination against one lineage provides limited or no cross-protection against B strains from the alternate lineage [6-8]. This means that vaccine effectiveness is likely to be compromised during seasons when lineage-mismatched influenza B strains are prevalent. In a recent meta-analysis of randomized controlled trials reporting laboratory-confirmed influenza in children, adults, and elderly adults, in seven trials where the TIV was mismatched for influenza B strains, the vaccine efficacy was 52% (95% CI: 19, 72), whereas in five trials where the TIV was matched for influenza B, the vaccine efficacy was 77% (95% CI: 18, 94) [9].

During about half of the influenza seasons in the past decade, the recommended TIV was mismatched for the circulating influenza B lineage; between 2000 and 2011, the predominant influenza B viruses detected by US surveillance in 6 out of 11 influenza seasons were B lineage mismatched for the recommended TIV, and mismatch was observed in Europe during 4 out of 8 influenza seasons between 2003 and 2011 [5,10]. The use of quadrivalent vaccines including an influenza B strain of each lineage is likely to improve vaccine protection. In a modeling study conducted by the US Center for Disease Control and Prevention (CDC), based on the clinical burden of influenza illness and viral surveillance, the use of a quadrivalent vaccine rather than a TIV between 1999 and 2009 could have resulted in between 2200 and 970,000 fewer influenza illnesses, between 14 and 8200 fewer influenza-associated respiratory hospitalizations, and between 1 and 485 fewer influenza-related respiratory deaths in the US [11].

In February 2012, the World Health Organization (WHO) recommended strains for inclusion in quadrivalent vaccines for use in the 2012/2013 influenza season in the Northern Hemisphere, heralding a new era in influenza vaccination strategy [12]. In the same month, FluMist® (MedImmune), a live attenuated influenza vaccine for intranasal administration in people aged 2–49 years, became the first quadrivalent influenza vaccine formulation to gain approval in the US [13,14]. In this paper, we discuss a vaccine trial conducted in adults and elderly adults in stable health, which evaluated an established TIV for intramuscular administration (*Fluarix™*; GlaxoSmithKline Vaccines), formulated with an additional influenza B strain.

The Phase III, multinational study was randomized and partially-blind and assessed the immunogenicity, reactogenicity, and safety of the candidate inactivated quadrivalent split virion influenza vaccine (QIV) versus two TIVs containing a strain from each of the B lineages. The main aims were to demonstrate the immunological consistency of three QIV lots, to show the non-inferiority of hemagglutination inhibition (HI) antibody responses for QIV versus TIVs against shared influenza A and B strains, and to assess the superiority of HI antibody responses of QIV versus TIVs for the alternate B lineage.

## Methods

This Phase III, randomized, partially-blind, multinational study evaluated the immunogenicity, reactogenicity and safety of a candidate QIV versus TIV in adults aged ≥18 years. The study was conducted between October 2010 and June 2011 in Germany, Romania, Spain, Korea, Taiwan and the US.

Eligible subjects were aged ≥18 years and were in stable health without significant pulmonary, cardiovascular, hepatic or renal disease. Subjects were excluded if they had received any seasonal influenza vaccination within 6 months or any investigational product within 30 days before vaccination in this study. Other criteria for exclusion were history of Guillain Barré syndrome, hypersensitivity to a previous dose of influenza vaccine or its components, immunosupression, or receipt of immunoglobulins or blood products within 3 months before vaccination.

The study was conducted in accordance with the Good Clinical Practice guidelines, the Declaration of Helsinki and applicable local regulations. All study documents were approved by independent national or regional ethics committees or institutional review boards. All subjects provided written informed consent. Clintrials.gov NCT01204671.

### Vaccines and randomization

The study vaccines were developed and manufactured by GlaxoSmithKline (GSK), Dresden, Germany. The QIV candidate was a thimerosal-free, inactivated split virion vaccine containing two influenza A strains (A/California/7/2009 [H1N1] and A/Victoria/210/2009 [H3N2], an A/Perth/16/2009-like strain) and one influenza B strain (B/Brisbane/60/2008 [Victoria-lineage]) recommended for the influenza season 2010–2011 in the Northern Hemisphere, and one influenza B strain from the B/Yamagata lineage (B/Brisbane/3/2007, a B/Florida/4/2006 like strain previously recommended for the 2008–2009 influenza season). The TIVs contained B/Victoria (TIV-Vic) or TIV-B/Yamagata (TIV-Yam). TIV-Vic (*Fluarix*™, a trademark of the GlaxoSmithKline Vaccines) was a thimerosal-free, inactivated, split virion TIV containing the strains recommended for the influenza season 2010–2011 in the Northern Hemisphere. TIV-Yam differed from TIV-Vic only in the influenza B virus lineage (B/Brisbane/3/2007 [Yamagata-lineage]). The vaccines contained 15 μg of each hemagglutinin antigen, and were given as a 0.5 ml dose.

A randomization list was generated by the study sponsor using MATEX, a program developed for SAS® (Cary, NC, USA). Treatment allocation at each center was performed via an internet-based system; groups had an equal distribution of subjects aged 18–64 years versus ≥ 65 years and a minimization algorithm was used to account for center, and influenza vaccination in the previous season. Subjects were randomized 5:5:5:5:3 to receive QIV lot 1, 2, or 3, TIV-Vic, or TIV-Yam. All personnel and subjects were blind to the vaccine allocation of QIV or TIV-Vic, whereas TIV-Yam was open-label. Subjects in the QIV and TIV-Vic groups were followed-up for 6 months, whereas the study stopped at Day 21 for the TIV-Yam group so these subjects could choose to receive a licensed vaccine for the current season. Subjects received one dose of vaccine in the deltoid of the non-dominant arm.

### Assessments

#### Immunogenicity

Blood was collected in a subset of subjects for serological testing before vaccination (Day 0) and at Day 21 post-vaccination. Hemagglutination inhibition (HI) antibody titers against the vaccine strains were assessed at GlaxoSmithKline Vaccines central laboratory using validated assay methods as previously described [15].

Immunogenicity parameters were geometric mean titer (GMT), seroprotection rate (SPR; proportion with post-vaccination titer ≥ 1:40), seroconversion rate (SCR; proportion with antibody titer < 1:10 at baseline and with post-vaccination titer of ≥1:40, or pre-vaccination titer of ≥ 1:10 and a ≥ 4-fold post-vaccination increase in titer), and seroconversion factor (SCF; geometric mean of the ratio between pre-vaccination and post-vaccination reciprocal HI titers). Subjects with HI antibody titers of ≥ 1:10 were considered to be seropositive.

#### Reactogenicity and safety

Subjects used diary cards to record solicited injection site adverse events (pain, redness and swelling) and general adverse events (fatigue, fever, gastrointestinal symptoms, headache, joint pain, general muscle ache and shivering) during the 7-day post-vaccination period. Intensity of solicited symptoms was graded on a standard scale (0–3); Grade 1 symptoms were defined as those not interfering with normal activities and Grade 3 symptoms were defined as those preventing normal activities (Grade 3 redness and swelling: diameter > 100 mm; Grade 3 fever: temperature > 39°C [> 102.2°F]).

Unsolicited adverse events (AEs), serious adverse events (SAEs), and medically-attended adverse events (MAEs) were collected and were coded using the Medical Dictionary for Regulatory Activities. All solicited injection site symptoms were considered vaccination-related, and investigators judged causality with the vaccination for all other events.

### Objectives

The co-primary objectives were to evaluate for the study population overall: the lot-to-lot consistency of three QIV lots based on GMTs at Day 21 post-vaccination; the non-inferiority of GMTs and SCRs at Day 21 for QIV versus TIV-Vic and TIV-Yam against the shared strains, and the superiority of GMTs and SCRs at Day 21 for QIV versus TIV-Vic and TIV-Yam against the alternate-lineage B strains (i.e. QIV versus TIV-Vic against B/Yamagata, and QIV versus TIV-Yam against B/Victoria).

The secondary objectives were to describe: immunogenicity parameters in the population overall and stratified by age (18–64 years and ≥ 65 years); solicited adverse events (AEs) during the 7 day post-vaccinated period; unsolicited AEs during the 21-day post-vaccination period; SAEs and MAEs during the 6 months study period in the QIV and TIV-Vic group, and for 21 days post-vaccination in the open-label TIV-Yam group.

### Statistical analyses

The target sample size for the immunogenicity sub-cohort was 570 evaluable subjects, based on the global power to meet all co-primary objectives of at least 90%. A target sample of 1000 subjects per lot in the QIV group was based on the power to evaluate lot-to-lot consistency estimated using PASS, equivalence test of two means using differences (α = 5.0%) and applying the Zmin test principle. The sample size of 1000 subjects in the TIV-Vic group was based on the power for evaluating the non-inferiority and superiority objectives estimated using PASS, one-sided two-sample t-test for a difference of means (α = 2.5%; for HI antibody GMT ratios) and on the difference of proportions (α = 2.5%; for HI antibody SCRs). The total sample size of 4600 was determined to allow 3000 subjects in the QIV group, which would enable the detection of AEs occurring at a rate of 0.1%.

Adjusted GMTs were estimated using an ANCOVA model fitted on log10 transformed post-vaccination HI titer including treatment as fixed effect and baseline titer as a covariate. The SCR difference and the two-sided 95% CI of the SCR differences were computed after fitting a logistic regression on the SCR, including vaccine group as a fixed effect and baseline titer as a covariate. The co-primary confirmatory analyses were performed in the following order: Lot-to-lot consistency was based on adjusted GMT ratios for pairwise comparisons of QIV lots (lot 1/lot 2, lot 1/lot 3, lot 2/lot 3) for each strain; the pair with the largest GMT ratio for each strain was evaluated, and lot-to-lot consistency was demonstrated if the two-sided 95% CI limit was between 0.67 and 1.5 for all four strains. Non-inferior immunogenicity of QIV versus TIV for shared strains was demonstrated if the upper limit of the two-sided 95% CI for the adjusted GMT ratio of TIV/QIV did not exceed 1.5, *and* the upper limit of the two-sided 95% CI for the SCR difference (TIV minus QIV) did not exceed 10.0. Superior immunogenicity of QIV versus TIV for the alternate-lineage B strain was demonstrated if the lower limit of the two-sided 95% CI on the adjusted GMT ratio (QIV/TIV-Vic and QIV/TIV-Yam) was greater than 1.0, *and* the lower limit of the two-sided 95% CI for the SCR difference (QIV minus TIV-Vic or TIV-Yam) was greater than 0.

Immunogenicity parameters were described with 95% CIs. Immunogenicity analyses were performed on the per-protocol immunogenicity sub-cohort including subjects who met the eligibility criteria and complied with the protocol (per-protocol immunogenicity cohort), and whom were allocated to the immunogenicity sub-cohort (the first 600 subjects randomized in each group account the age stratification and the minimization factors), and for whom data were available at the evaluation time point.

Solicited and unsolicited AEs were tabulated with 95% CIs. Reactogenicity and safety analyses were performed on the total vaccinated cohort.

## Results

**Figure 1:**
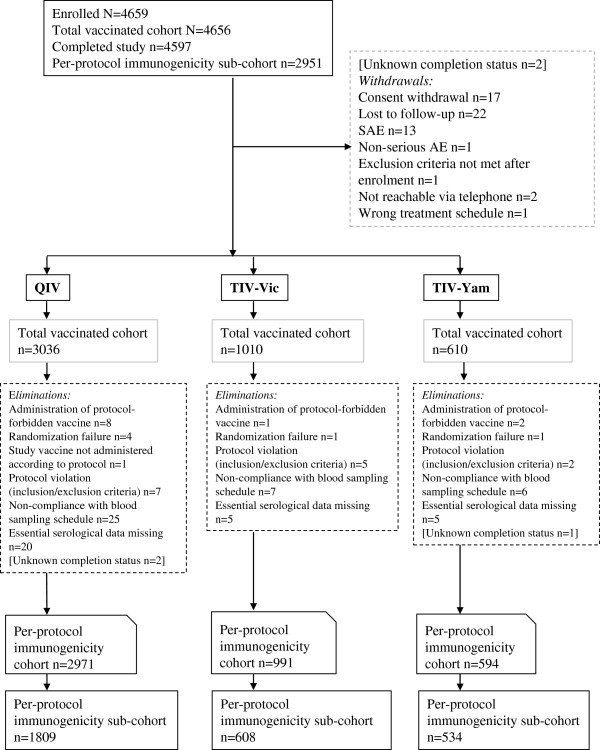
**Subject flow. ***Footnote:* QIV, inactivated quadrivalent influenza vaccine; TIV-Vic, inactivated trivalent influenza vaccine Victoria lineage B strain; TIV-Yam, inactivated trivalent influenza vaccine Yamagata lineage B strain; SAE, serious adverse event.

A total of 4659 subjects were enrolled, of which 4656 subjects were vaccinated: Germany n = 651, Romania n = 650, Spain n = 672, Korea n = 832, Taiwan n = 400 and the US n = 1451. A total of 4597 subjects completed the study (Figure 1). The reasons for withdrawals and exclusion are shown in Figure 1. The demographic characteristics were balanced across all study groups (Table 1). A review of the reported medical history revealed that cardiovascular diseases (excluding hypertension), diabetes and chronic respiratory diseases (reported by 17%, 14% and 10% of the subjects respectively) were the most frequently reported risk factors for influenza disease complications. In each group, about 80% of subjects had received at least one seasonal influenza vaccine during the previous three seasons.

**Table 1:** Demographic and clinical characteristics in the total vaccinated cohort

	**QIV**	**TIV-Vic**	**TIV-Yam**
	**N = 3036**	**N = 1010**	**N = 610**
Mean age, years (SD; median; range)	57.9 (17.7; 64.0; 18.0–92.0)	58.1 (17.8; 64.0; 18.0–92.0)	58.1 (17.9; 65.0; 18.0–90.0)
Sub-groups: mean age, years (SD, median)	43.5 (13.58; 44.0)	43.7 (13.84; 44.0)	43.5 (14.01; 46.0)
*18–64 years*	72.3 (5.45; 71.0)	72.5 (5.53; 71.0)	72.4 (5.39; 72.0)
*≥65 years*			
Male, n (%)	1291 (42.5)	462 (45.7)	267 (43.8)
Hispanic/Latino ethnicity, n (%)	134 (4.4)	47 (4.7)	25 (4.1)
Not Hispanic/Latino ethnicity, n (%)	2902 (95.6)	963 (95.3)	585 (95.9)
Geographic ancestry	2078 (68.4)	699 (69.2)	414 (67.9)
*European heritage / Caucasian*	22 (0.7)	7 (0.7)	4 (0.7)
*Arabic/north American heritage / Caucasian*	805 (26.5)	270 (26.7)	162 (26.5)
*Asian*	106 (3.5)	26 (2.6)	21 (3.4)
*African heritage / African American*	6 (0.2)	2 (0.2)	3 (0.5)
*American Indian or native Alaskan*	2 (0.1)	0	0
*Native Hawaiian or other pacific islander*	17 (0.6)	6 (0.6)	6 (1.0)
*Other*			
Medical history, n (%)	533 (17.56)	153 (15.15)	106 (17.38)
*Cardiovascular disease*^*†*^	457 (15.05)	129 (12.77)	89 (14.59)
*Diabetes*	293 (9.65)	104 (10.30)	57 (9.34)
*Chronic respiratory disease*	117 (3.85)	39 (3.86)	26 (4.26)
*Cancer*	76 (2.50)	17 (1.68)	13 (2.13)
*Cerebrovascular disease*	60 (1.98)	23 (2.28)	13 (2.13)
*Chronic hepatic disease*	43 (1.42)	11 (1.09)	6 (0.98)
*Chronic renal disease*			
Received seasonal influenza vaccination in at least one of previous three seasons, n (%)	2395 (78.9)	788 (78.0)	483 (79.2)
Received A/H1N1pdm09 vaccination during the previous season	848 (27.9)	289 (28.6)	165 (27.0)

*QIV* inactivated quadrivalent influenza vaccine, *SD* standard deviation, *TIV-Vic* inactivated trivalent influenza vaccine Victoria lineage B strain, *TIV-Yam* inactivated trivalent influenza vaccine Yamagata lineage B strain, ^†^Excluding hypertension.

### Immunogenicity

#### Confirmatory analyses

The limits of the two-sided 95% CI for the largest adjusted GMT ratios at Day 21 among the three lots of QIV were between 0.67 and 1.5 for each of the four strains, and therefore the criteria for lot-to-lot consistency were met (Table 2).

**Table 2:** Lot-to-lot consistency of QIV lots based on HI-assay based GMTs at Day 21 in the per-protocol immunogenicity sub-cohort

	**Min**^**†**^		**Max**^**‡**^		**Adjusted GMT ratio**
	**n**	**Adjusted GMT**	**n**	**Adjusted GMT**	**Min/Max**^**¶**§^**(95% CI)**
A/California/7/2009 (H1N1)	600	196.5	599	209.0	0.94 (0.80–1.10)
A/Victoria/210/2009 (H3N2)	600	306.8	599	330.6	0.93 (0.81–1.06)
B/Brisbane/60/2008 (Victoria lineage)	600	410.7	599	396.7	1.04 (0.93–1.15)
B/Brisbane/3/2007 (Yamagata lineage)	600	605.0	599	599.0	1.01 (0.90–1.13)

^†^Lot with lowest GMT; ^‡^Lot with highest GMT; ^¶^Pair with the largest GMT ratio for each strain from pairwise comparisons was assessed and consistency was demonstrated if the 2-sided 95% CI limit was between 0.67 and 1.5 for all four strains; ^§^for each strain, lot 1/lot 2.

*CI* confidence interval, *GMT* geometric mean titer, *HI* hemagglutination inhibition, *QIV* quadrivalent inactivated influenza vaccine.

Non-inferior immunogenicity at Day 21 based on adjusted GMT ratio and SCR difference was shown for the QIV candidate versus the TIV pooled for influenza A strains, and versus TIV-Vic for B/Victoria, and TIV-Yam for B/Yamagata (shared strains) (Table 3). Superior immunogenicity at Day 21 based on adjusted GMT ratio and SCR difference was shown for the QIV candidate versus TIV-Vic for B/Yamagata and versus TIV-Yam for B/Victoria (alternate-lineage B strains) (Table 4).

**Table 3:** Non-inferiority of QIV versus TIVs against shared strains according to HI-assay based GMT and SCR at Day 21 in the per-protocol immunogenicity sub-cohort

**Vaccine antigen**	**Adjusted GMT**		**Adjusted GMT ratio (95% CI)**^**†**^
	**TIV-Vic + TIV-Yam, N = 1135**	**QIV, N = 1801**	**TIV-Vic + TIV-Yam/QIV**
A/California/7/2009 (H1N1)	214.8	201.6	1.07 (0.96, 1.18)
A/Victoria/210/2009 (H3N2)	312.2	318.5	0.98 (0.90, 1.07)
B/Brisbane/60/2008	**TIV-Vic, N = 605**	**QIV, N = 1801**	**TIV-Vic/QIV**
(Victoria lineage)	395.3	404.2	0.98 (0.9, 1.07)
B/Brisbane/3/2007	**TIV-Yam, N = 530**	**QIV, N = 1801**	**TIV-Yam/QIV**
(Yamagata lineage)	584.7	600.8	0.97 (0.89, 1.07)
	**Number seroconverted (SCR)**		**SCR difference (95% CI)**^**‡**^
	**TIV-Vic + TIV-Yam, N = 1135**	**QIV, N = 1801**	**TIV-Vic + TIV-Yam minus QIV**
A/California/7/2009 (H1N1)	892 (78.6%)	1396 (77.5%)	1.08 (−2.03, 4.11)
A/Victoria/210/2009 (H3N2)	769 (67.8%)	1287 (71.5%)	−3.71 (−7.15, −0.30)
B/Brisbane/60/2008	**TIV-Vic, N = 605**	**QIV, N = 1801**	**TIV-Vic minus QIV**
(Victoria lineage)	335 (55.4%)	1046 (58.1%)	−2.71 (−7.29, 1.83)
B/Brisbane/3/2007	**TIV-Yam, N = 530**	**QIV, N = 1801**	**TIV-Yam minus QIV**
(Yamagata lineage)	313 (59.1%)	1112 (61.7%)	−2.69 (−7.47, 2.01)

^**†**^Non-inferiority was demonstrated if the lower limit of the 95% CI was ≤1.5; ^**‡**^Non-inferiority was demonstrated if the lower limit of the 95% CI ≤10.0.

*CI* confidence interval, *GMT* geometric mean titer, *HI* Hemagglutination inhibition, *SCR* seroconversion rate (proportion with pre-vaccination titer <1:10 and a post-vaccination titer ≥1:40, or a pre-vaccination titer ≥1:10 and at least a four-fold increase in post-vaccination titer), *TIV* trivalent inactivated influenza vaccine, *Vic* Victoria lineage B strain, *Yam* Yamagata lineage B strain, *QIV* quadrivalent inactivated influenza vaccine.

**Table 4:** Superiority of QIV versus TIVs against alternate lineage B strains according to HI-assay based GMT and SCR at Day 21 in the per-protocol cohort for immunogenicity

**Vaccine antigen**	**TIV-Vic**	**TIV-Yam**	**QIV**	**Superiority analysis**
	**N = 605**	**N = 530**	**N = 1081**	
	**Adjusted GMT**			**Adjusted GMT ratio (95% CI)**^**†**^
B/Brisbane/3/2007 (Yamagata lineage)	387.7	–	601.2	**QIV/TIV-Vic**
				1.55 (1.41, 1.70)
B/Brisbane/60/2008 (Victoria lineage)	–	259.4	403.5	**QIV/TIV-Yam**
				1.56 (1.42, 1.70)
	**Number seroconverted (SCR)**			**SCR difference (95% CI)**^**‡**^
B/Brisbane/3/2007 (Yamagata lineage)	276 (45.6%)	–	1112 (61.7%)	**QIV minus TIV-Vic**
				16.12% (11.54, 20.65)
B/Brisbane/60/2008 (Victoria lineage)	–	252 (47.5%)	1046 (58.1%)	**QIV minus TIV-Yam**
				10.53% (5.70, 15.33)

^†^Superiority was demonstrated if the lower limit of the 95% CI was > 1.0; ^**‡**^Superiority was demonstrated if the lower limit of the 95% CI was > 0%.

*CI* confidence interval, *GMT* geometric mean titer, *HI* Hemagglutination inhibition, *SCR* seroconversion rate (proportion with pre-vaccination titer < 1:10 and a post-vaccination titer ≥ 1:40, or a pre-vaccination titer ≥ 1:10 and at least a four-fold increase in post-vaccination titer), *TIV-Vic* inactivated trivalent influenza vaccine Victoria lineage B strain, *TIV-Yam* inactivated trivalent influenza vaccine Yamagata lineage B strain, *QIV* inactivated quadrivalent influenza vaccine.

#### Descriptive immunogenicity

The QIV candidate was highly immunogenic overall with HI antibody-based SCRs of 77.5% and 71.5% against A/H1N1 and A/H3N2, respectively, and 58.1% and 61.7% against B/Victoria and B/Yamagata, respectively (Table 5). In the TIV groups overall, SCRs against influenza A strains were 65.8–80.2%, and against B/Victoria and B/Yamagata were 55.4% and 45.6%, respectively for TIV-Vic, and 47.5% and 59.1% respectively for TIV-Yam (Table 5). QIV elicited more than a 1.5-fold higher mean HI antibody responses over each TIV control for the influenza B strain from the alternate lineage, which translated into an absolute SCR difference of at least 10.0%.

**Table 5:** Descriptive immunogenicity based on HI antibody titers in the per-protocol cohort for immunogenicity

**Parameter**	**Vaccine**	**Day**	**N**	**A/California/7/2009 (H1N1)**	**A/Victoria/210/2009 (H3N2)**	**B/Brisbane/60/2008 (Victoria)**	**B/Brisbane/3/2007 (Yamagata)**
**GMTs, value (95% CI)**	QIV	Day 0	1801	14.7	34.0	73.8	101.4
				(13.8, 15.6)	(31.8, 36.3)	(69.1, 78.8)	(94.5, 108.8)
		Day 21	1809	201.1	314.7	404.6	601.8
				(188.1, 215.1)	296.8, 333.6)	(386.6, 423.4)	(573.3, 631.6)
	TIV-Vic	Day 0	605	15.6	38.1	73.6	100.9
				(14.1, 17.3)	(34.1, 42.7)	(65.5, 82.8)	(89.3, 113.9)
		Day 21	608	218.4	298.2	393.8	386.6
				(194.2, 245.6)	(268.4, 331.3)	(362.7, 427.6)	(351.5, 425.3)
	TIV-Yam	Day 0	530	14.4	35.7	71.7	99.8
				(12.9, 16.0)	(31.6, 40.3)	(63.4, 81.0)	(87.7, 113.5)
		Day 21	534	213.0	340.4	258.5	582.5
				(187.6, 241.9)	(304.3, 380.9)	(234.6, 284.8)	(534.6, 634.7)
**SPR, % (95% CI)**	QIV	Day 0	1801	28.5%	53.6%	79.0%	83.0%
				(26.5, 30.7)	(51.2, 55.9)	(77.1, 80.9)	(81.1, 84.7)
		Day 21	1809	91.3%	96.8%	98.8%	99.1%
				(89.9, 92.5)	(95.9, 97.6)	(98.2, 99.3)	(98.5, 99.5)
	TIV-Vic	Day 0	605	27.6%	58.3%	78.8%	82.1%
				(24.1, 31.4)	(54.3, 62.3)	(75.4, 82.0)	(78.9, 85.1)
		Day 21	608	91.8%	95.9%	98.5%	97.9%
				(89.3, 93.8)	(94.0, 97.3)	(97.2, 99.3)	(96.4, 98.9)
	TIV-Yam	Day 0	530	26.2%	53.8%	77.7%	83.2%
				(22.5, 30.2)	(49.4, 58.1)	(74.0, 81.2)	(79.7, 86.3)
		Day 21	534	92.7%	96.8%	96.1%	99.6%
				(90.2, 94.8)	(95.0, 98.1)	(94.1, 97.5)	(98.7, 100)
**SCR, % (95%)**	QIV	Day 21	1801	77.5%	71.5%	58.1%	61.7%
				(75.5, 79.4)	(69.3, 73.5)	(55.8, 60.4)	(59.5, 64.0)
	TIV-Vic	Day 21	605	77.2%	65.8%	55.4%	45.6%
				(73.6, 80.5)	(61.9, 69.6)	(51.3, 59.4)	(41.6, 49.7)
	TIV-Yam	Day 21	530	80.2%	70.0%	47.5%	59.1%
				(76.5, 83.5)	(65.9, 73.9)	(43.2, 51.9)	(54.7, 63.3)
**SCF, value (95%)**	QIV	Day 21	1801	13.69	9.28	5.48	5.93
				(12.70, 14.76)	(8.64, 9.96)	(5.12, 5.85)	(5.53, 6.36)
	TIV-Vic	Day 21	605	13.92	7.84	5.37	3.84
				(12.23, 15.84)	(6.93, 8.88)	(4.75, 6.06)	(3.42, 4.30)
	TIV-Yam	Day 21	530	14.88	9.52	3.60	5.84
				(12.91, 17.16)	(8.33, 10.89)	(3.25, 3.98)	(5.13, 6.65)

*CI* confidence interval, *HI* hemagglutination-inhibition, *GMT* geometric mean titer, *SPR* seroprotection rate, *SCR* seroconversion rate, *SCF* seroconversion factor, *QIV* inactivated quadrivalent influenza vaccine, *TIV-Vic* inactivated trivalent influenza vaccine Victoria lineage B strain, *TIV-Yam* inactivated trivalent vaccine Yamagata lineage B strain.

SPR defined as proportion of subjects with HI antibody titers ≥ 1:40; SCR defined as proportion of subjects with a pre-vaccination HI antibody titer < 1:10 and post-vaccination HI antibody titer ≥ 1:40, or subjects with at least a 4-fold increase in the post-vaccination HI antibody titer; SCF defined as the geometric mean of the within subject ratios of reciprocal HI antibody titers for post-vaccination versus pre-vaccination.

**Figure 2:**
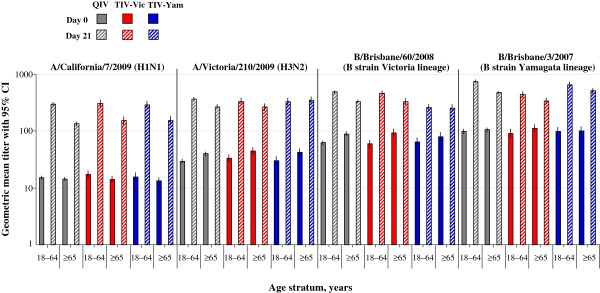
**HI antibody GMTs stratified by age (per-protocol immunogenicity sub-cohort). ***Footnote:* CI, confidence interval; GMT, geometric mean titer; QIV, inactivated quadrivalent influenza vaccine; TIV-Vic, inactivated trivalent influenza vaccine Victoria lineage B strain; TIV-Yam, inactivated trivalent influenza vaccine Yamagata lineage B strain.

**Figure 3:**
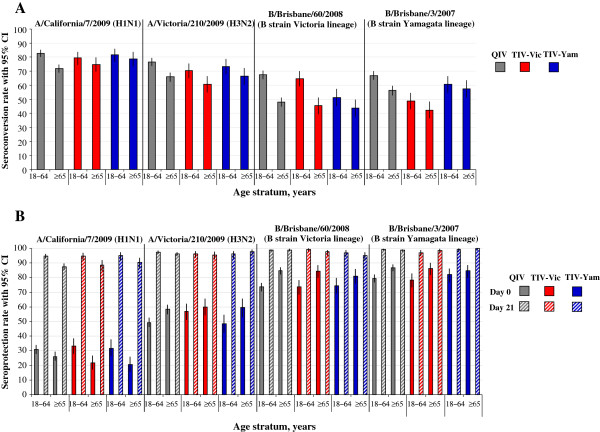
**HI antibody stratified by age (per-protocol immunogenicity sub-cohort). ***Footnote: ***(A)**. seroconversion rate, **(B)**. seroprotection rate. CI, confidence interval; QIV, Inactivated quadrivalent influenza vaccine; TIV-Vic, inactivated trivalent influenza vaccine Victoria lineage B strain; TIV-Yam, inactivated trivalent influenza vaccine Yamagata lineage B strain. Seroconversion rate defined as the proportion with antibody titer < 1:10 at baseline and with post-vaccination titer of ≥ 1:40, or pre-vaccination titer of ≥ 1:10 and a ≥ 4-fold post-vaccination increase in titer; Seroprotection rate defined as defined as proportion with post-vaccination titer ≥ 1:40.

Age-stratified GMTs are shown in Figure 2, and age-stratified SCRs and SPRs are shown in Figure 3. Age stratified data showed that the HI antibody response appeared to decrease with advancing age. The post-vaccination HI antibody GMTs across all groups for all vaccine strains were higher in subjects aged 18–64 years (ranged between 294.3 and 749.1) compared with subjects aged ≥ 65 years (ranged between 133.5 and 513.2). The post-vaccination HI antibody GMT for the alternate-lineage B strain was 436.4 for TIV-Vic and 259.9 for TIV-Yam in subjects 18–64 years, and 339.5 and 257.0, respectively, in subjects aged ≥ 65 years. The SCRs for all vaccine strains across all study groups were 60.5 –82.7% in subjects aged 18–64 years, and 45.4%–78.8% in subjects aged ≥ 65 years. The SCRs for the alternate-lineage B strain was 48.7% for TIV-Vic and 51.3% for TIV-Yam in subjects 18–64 years, and 42.3% and 43.6%, respectively in subjects aged ≥ 65 years. In the QIV group, the SCRs for all vaccine strains were 66.9–82.7% in subjects aged 18–64 years, and 48.0–71.9% in subjects aged ≥ 65 years.

### Reactogenicity and safety

**Figure 4:**
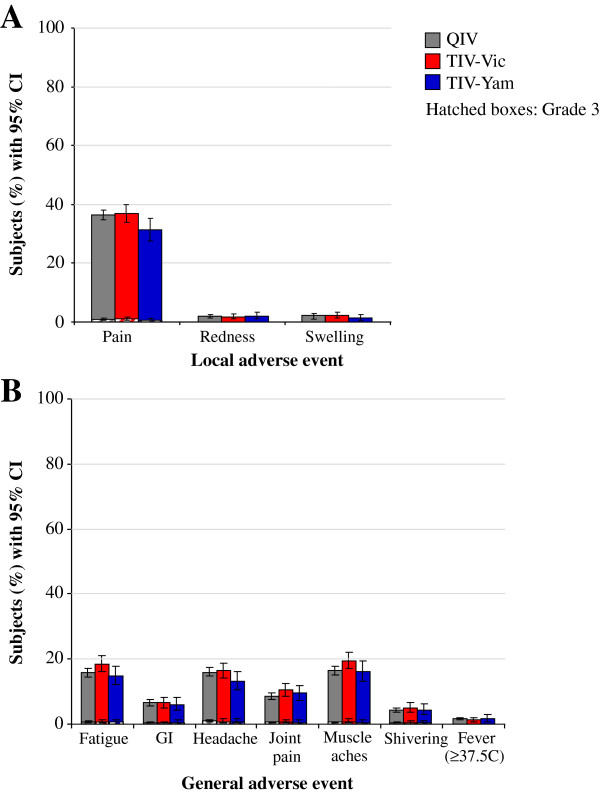
**Solicited adverse events (total vaccinated cohort). ***Footnote: ***(A)**. local adverse events, **(B)**. general adverse events. CI, confidence interval; GI, gastrointestinal; QIV, Inactivated quadrivalent influenza vaccine; TIV-Vic, inactivated trivalent influenza vaccine Victoria lineage B strain; TIV-Yam, inactivated trivalent influenza vaccine Yamagata lineage B strain.

Reactogenicity during the 7-day post-vaccination period is shown in Figure 4. Injection site pain was the most frequent local symptom, and was reported by 36.4%, 36.8% and 31.3% of the QIV, TIV-Vic, and TIV-Yam groups respectively. Grade 3 solicited injection site symptoms were reported for ≤ 1.2% of subjects in any group. In the QIV, TIV-Vic and TIV-Yam groups, the most frequent general symptoms were fatigue (15.8%, 18.4% and 14.8%, respectively), headache (15.9%, 16.4% and 13.2%, respectively) and muscle ache (16.4%, 19.4% and 16.1%, respectively). Grade 3 solicited general symptoms were reported in < 1.0% of subjects in any group.

An overview of unsolicited AEs and MAEs is shown in Table 6. Overall during the 21-day post-vaccination follow-up period (Day 0 to Day 20) in the QIV, TIV-Vic, and TIV-Yam groups, respectively, 379 (12.5%), 138 (13.7%) and 92 (15.1%), subjects reported at least 1 unsolicited AE. Grade 3 AEs were reported by ≤ 1.3% of subjects. Nasopharyngitis and cough were the most frequently reported unsolicited AEs in each group (1.4%–1.7%). Overall, ≤ 2.6% of subjects reported AEs that were considered by the investigator to be related to vaccination. During the 21-day post-vaccination period, in the QIV, TIV-Vic, and TIV-Yam groups, respectively, 193 (6.4%), 60 (5.9%) and 47 (7.7%), subjects reported at least 1 MAE. In the QIV group, the most frequent MAEs during the 21 day post-vaccination period were nasopharyngitis (0.8%) and cough (0.5%), in the TIV-Vic group were nasopharyngitis (0.8%) and sinusitis (0.4%), and in the TIV-Yam group were nasopharyngitis (0.8%) and urinary tract infection (0.7%). During the 6 month follow-up, MAEs were reported by 688 (22.7%) and 216 (21.4%) subjects in the QIV group and in the TIV-Vic group, respectively.

**Table 6:** Overview of unsolicited AEs and MAEs in the total vaccinated cohort

	**QIV**	**TIV-Vic**	**TIV-Yam**
	**N = 3036**	**N = 1010**	**N = 610**
**Adverse events Day 0–20, n (%)**			
Subjects with ≥ 1 event	379 (12.5)	138 (13.7)	92 (15.1)
Subjects with ≥ 1 Grade 3 event	39 (1.3)	7 (0.7)	2 (0.3)
Subjects with ≥ 1 event related to vaccination^†^	64 (2.1)	26 (2.6)	14 (2.3)
No. of events by MedDRA preferred term	558	195	125
No. of Grade 3 events by MedDRA preferred term	52	8	2
No. of events by MedDRA preferred term related to vaccination^†^	89	38	16
**Medically-attended adverse events Day 0–20, n (%)**			
Subjects with ≥ 1 event	193 (6.4)	60 (5.9)	47 (7.7)
No. of events by MedDRA preferred term	250	75	63
**Medically-attended adverse events Day 0–180, n (%)**			
Subjects with ≥ 1 event	688 (22.7)	216 (21.4)	NA*
No. of events by MedDRA preferred term	1151	379	NA*

*AE* adverse event, *MAE* medically-attended adverse event, *QIV* inactivated quadrivalent influenza vaccine, *TIV-Vic* inactivated trivalent influenza vaccine Victoria lineage B strain, *TIV-Yam* inactivated trivalent influenza vaccine Yamagata lineage B strain, *MedDRA* Medical Dictionary for Regulatory Activities, *NA* not applicable, ^†^Based on investigator’s assessment of causality; *TIV-YAM group was followed up until Day 21.

During the 21-day post-vaccination period in the QIV, TIV-Vic, and TIV-Yam groups, respectively, 16 (0.5%), 6 (0.6%), and 1 (0.2%) subjects experienced at least 1 SAE. During the 6 month follow-up, 98 SAEs were reported by 70 subjects (2.3%) in the QIV group and 27 SAEs were reported by 26 subjects (2.6%) in the TIV-Vic group. The most frequent SAE(s) in the QIV group were myocardial infarction and cerebrovascular accident (5 reports each; 0.2%), in the TIV-Vic group was pneumonia, cerebrovacular accident and nephrolithiasis (2 reports each; 0.2%), and in the TIV-Yam group the only SAE was arteriosclerosis. Twelve subjects, all aged ≥ 65 years, died during the study (Table 7). There were 9 deaths in the QIV group (2 cardiac disorders, 1 neoplasm, 1 intestinal infarction, 2 sudden deaths, 1 hepatic coma, 1 cerebrovascular accident and 1 pulmonary hypertension). There were 3 deaths in the TIV-Vic group, all due to cardiac disorders. None of the deaths were considered by the investigator to be related to vaccination.

**Table 7:** Description of fatal SAEs in the total vaccinated cohort

**Vaccine group**	**Subject code**	**Country**	**Age at onset, years**	**Gender**	**MedDRA Preferred term**	**Day of onset post-vaccination**
QIV N = 3036	461	Taiwan	85	M	Sudden death	86
	2140	Republic of Korea	85	F	Cardiac failure congestive Myocardial infarction	131
	3023	Germany	84	F	Death	62
	4373	Romania	81	F	Cardiopulmonary failure	87
					Intestinal infarction	86
	6609	US	71	M	Pulmonary hypertension	162
	7347	US	72	F	Myocardial infarction	35
	987	Republic of Korea	68	F	Coma hepatic	95
	5468	Spain	73	M	Cerebrovascular accident	191
	6594	US	71	F	Small cell lung cancer stage unspecified	51
TIV-Vic N = 1010	3735	Romania	86	M	Cardiac arrest	75
					Myocardial infarction	
	4362	Romania	69	M	Cardio-respiratory arrest	97
	5518	Spain	69	M	Cardiac disorder	15

*SAE* serious adverse event, *QIV* inactivated quadrivalent influenza vaccine, *TIV-Vic* inactivated trivalent influenza vaccine Victoria lineage B strain, *MedDRA* Medical Dictionary for Regulatory Activities, *F* female, *M* male.

## Discussion

This Phase III, randomized study showed that HI antibody responses of a candidate QIV were non-inferior for shared influenza A and B vaccine strains, and superior for alternate-lineage influenza B strains compared with TIV in adults aged ≥ 18 years in stable health. Manufacturing consistency of HI antibody responses was also demonstrated for three QIV vaccine lots. The candidate QIV had an acceptable reactogenicity and safety profile which was consistent with that of TIV. These results show that the candidate QIV provided superior immunogenicity for the additional B strain compared with TIV, without interfering with antibody responses to the three TIV antigens. This suggests that the candidate QIV is a viable alternative to TIV for use in adults aged ≥ 18 years, and could potentially improve protection against influenza B.

Influenza vaccines offer the greatest protection against influenza strains matched to the vaccine strains, and when there is a B-lineage mismatch, vaccine protection is reduced [8,9,16]. Indeed, the control TIV used in our study has been previously shown to be associated with vaccine efficacy of 67% (95% CI: 52, 77) in adults aged 18–64 years against vaccine-matching, culture-confirmed influenza, whereas in another study conducted during a season when viral circulation of influenza A was low, and the vaccine was mismatched to the prevalent influenza B virus, vaccine efficacy was not significant versus placebo [17,18]. A QIV containing strains from both B lineages could eliminate the risk of B lineage mismatch and be expected to provide improved protection against influenza B [11].

The candidate QIV in our study had superior antibody responses for the additional B strain compared with each TIV in which the strain was absent. However, whenever additional antigens are added to a vaccine, it is necessary to ensure that new antigens do not interfere with the immunogenicity of the existing vaccine antigens. For the candidate QIV in our study, the absence of immunologic interference was established by demonstrating non-inferiority between HI antibody responses elicited by the candidate QIV and TIV-Vic and TIV-Yam for the shared TIV strains. We showed that QIV elicited strong HI antibody responses, with SCRs of 77.5% and 71.5% against A/H1N1 and A/H3N2, respectively, 58.1% against B/Victoria, and 61.7% against B/Yamagata.

Influenza B is thought to disproportionately affect children and young people, and in adults, particularly elderly adults, influenza A (notably H3N2) is reported to be associated with higher rates of influenza-related complications and deaths than influenza B [19]. Nonetheless, influenza B epidemics in adults about every 2–4 years, and infection with influenza B virus confers an important risk of severe illness and hospitalization [19-22]. Moreover, in a modeling study of viral respiratory disease among hospitalized patients based on data from the UK, whereas influenza A was found to represent the highest ranking burden based on disability-adjusted-life-years among patients aged 16–64 and > 65 years, influenza B ranked fourth and second in the younger and older age groups, respectively [23]. A major finding of the modeling study was that the burden of influenza B disease was 100-fold higher in the > 65 years group than the 18–64 years group, whereas the corresponding increase between young and old for influenza A was about 50-fold [23].

In our sub-group analysis by age, although immune responses were generally lower for subjects aged ≥ 65 years than those aged 18–65 years, the QIV candidate was immunogenic for all four vaccine strains, and HI antibody responses against all strains fulfilled CBER immunogenicity acceptance criteria in both age strata [24]. TIV also elicited robust antibody responses against the three vaccine strains. The responses against alternate-lineage B strains were lower than the response observed for the B strain contained in the vaccine. The proportion of subjects overall with pre-vaccination antibody titers of ≥1:40 against B/Yamagata in the TIV-Vic group was 82.1% and against B/Victoria in the TIV-Yam group was 77.7%. High baseline antibody levels to influenza B strains from both lineages are likely to have facilitated booster responses to the B lineage absent from each TIV control group. Nevertheless, cross reactive HI antibody may not correlate with protection, as very high rates of breakthrough infections have been observed in elderly populations exposed to vaccine-lineage mismatched influenza B viruses even when vaccination elicited good cross-reactive HI booster responses [25].

We previously evaluated the immunogenicity of the QIV candidate in adults aged 18–60 years in a Phase II trial conducted in the Czech Republic between July and August 2008, and the GMT ratios confirming superior immunogenicity for the B strain unique to the QIV candidate were notably higher than in the current Phase III study [Phase II study under review for publication]. In the Phase II study, the GMT ratio for QIV/TIV-Vic against B/Yamagata was 4.08 (95% CI: 3.26, 5.11) compared with 1.55 (95% CI: 1.41, 1.70) in the Phase III study; however, in the Phase II study, the pre- and post-vaccination GMTs against B/Yamagata were 19.2 and 43.4 (TIV-Vic) and 19.6 and 179.1 (QIV), compared with 100.9 and 386.6 (TIV-Vic) and 101.4 and 601.8 (QIV) in the Phase III study. The difference in GMT ratio observed in the Phase II and Phase III studies likely reflects differences between the study populations regarding their recent prior exposure to influenza B viruses. For example, in the Phase III study, about 60% and 70% of participants respectively received TIV in 2008–2009 and 2009–2010, which sequentially contained the same B/Yamagata and B/Victoria strain contained in the present QIV candidate. Indeed, the superior immunogenicity of the QIV candidate versus TIV for the additional B strain would likely vary year to year, and as such, potentially improved protection may also vary. Nevertheless, this Phase III study provides a conservative estimate of superior immunogenicity, given the relatively high baseline antibody levels against each B lineage.

Because the QIV contains 60 μg of influenza antigen, it was possible that reactogenicity could be higher than with the TIV, which contains 45 μg of antigen. In this study, however, the reactogenicity and safety profile of the QIV candidate was consistent with the TIVs. None of the SAEs observed during the six month follow-up were considered by the investigators to be related to the study vaccines. The results suggest that the inclusion of an additional 15 μg of antigen in the QIV candidate did not compromise safety compared with TIV.

A limitation of the study was a concern regarding the compliance with Good Clinical Practice at a single study site in Romania which enrolled 45 subjects in the trial. A sensitivity analysis excluding data from this site was performed which did not impact study conclusions; therefore, data from this site were not excluded from the final analyses.

## Conclusions

In conclusion, because TIVs contain only one influenza B strain, vaccine mismatch for the alternate-lineage B strain is frequent, resulting in reduced vaccine protection. In this study of adults and elderly adults, we showed that a QIV candidate versus TIV provided non-inferior immunogenicity for shared influenza A and B strains, while also providing superior immunogenicity for the additional B strain. The reactogenicity and safety profile of the QIV candidate was consistent with TIV. These results suggest that a switch from TIV to QIV is a viable approach to influenza vaccination with the aim of eliminating influenza B lineage mismatch to potentially improve protection against influenza B.

## Abbreviations

AE: Adverse event; ANCOVA: Analysis of covariance; CI: Confidence Interval; GMT: Geometric Mean Titer; GSK: GlaxoSmithKline; HI: Hemagglutination Inhibition; LL: Lower limit; QIV: Inactivated quadrivalent influenza vaccine; SAEs: Serious Adverse Events; SCF: Seroconversion factor; SCR: Seroconversion Rate; SPR: Seroprotection Rate; TIV-Vic: Inactivated trivalent influenza vaccine Victoria lineage B strain; TIV-Yam: Inactivated trivalent influenza vaccine Yamagata lineage B strain; TVC: Total vaccinated cohort; UL: Upper limit.

## Competing interest

All participating institutions received compensation for study involvement. BL Innis, V Jain, C Claeys, M Peeters and Y Feng are employees of the GlaxoSmithKline group of companies. C Claeys, M Peeters, BL Innis and V Jain report ownership of stock options. D Kieninger reports payments received from GlaxoSmithKline group of companies and Wyeth for development of educational presentations, from GlaxoSmithKline group of companies and Novartis for travel/accommodation/meeting support, and grants from GlaxoSmithKline group of companies, Pfizer, Novartis and Sanofi-Pasteur. JM Bayas reports payments received from GlaxoSmithKline group of companies for board membership and lectures including services on speaker bureau. E Sheldon is an employee of Miami Research Associates. M Esen institution received a grant for the founding of an independent junior research group by the Bundesministerium für Forschung und Bildung. EJ Gabor reports payment received as speaker on Meningitis and tick-borne encephalitis for general practitioners and received payment from GlaxoSmithKline group of companies for travel/accommodation/meeting support. CJ Yu, C Alvarez, JL Fernandez Roure, S Narejos Perez and WY Lin declare having no conflict of interest.

## Authors’ contributions

All authors participated in the implementation of the study including substantial contributions to conception and design, the gathering of the data, or analysis and interpretation of the data. BLI, VJ, MP, and CC led the clinical team at GlaxoSmithKline Vaccines and were involved in all phases of the study. YF conducted the statistical analysis. DK, ES, WYL, CJY, JMB, EJG, ME, JLRF, SNP, and CAS coordinated the study at the investigator site. All authors were involved in the drafting of the article or revising it critically for important intellectual content, and approved the final version before submission.

## Pre-publication history

The pre-publication history for this paper can be accessed here:

http://www.biomedcentral.com/1471-2334/13/343/prepub

## Trade mark statement

*Fluarix*™ is a trade mark of the GlaxoSmithKline group of companies.
